# Occlusive dressing-induced secretomes influence the migration and proliferation of mesenchymal stem cells and fibroblasts differently

**DOI:** 10.1186/s40001-018-0357-2

**Published:** 2018-12-26

**Authors:** Michael K. Cerny, Ursula Hopfner, Manuela Kirsch, Elisabeth-Maria Haas, Fan Wu, Riccardo Giunta, Hans-Guenther Machens, Dominik Duscher, Holger Erne, Arndt F. Schilling

**Affiliations:** 1 Department for Plastic Surgery and Hand Surgery, Klinikum Rechts der Isar, Technical University Munich, Ismaninger Str.22, 81675 Munich, Germany; 2 Department for Hand Surgery, Plastic Surgery and Aesthetic Surgery, Ludwig-Maximilian-University Munich, Munich, Germany; 3 Department for Trauma Surgery, Orthopedic Surgery and Plastic Surgery, University Medical Center Goettingen, Goettingen, Germany

**Keywords:** Occlusive dressings, Fingertip injuries, MSC, Scar

## Abstract

**Background:**

Fingertip injuries treated with occlusive dressings (ODs) lead to nearly scar-free, functionally, and aesthetically pleasing results. We hypothesized that paracrine factors in the wound fluid (secretome) may influence migration and proliferation of mesenchymal stem cells (MSCs) and fibroblasts and modulate the wound-healing process.

**Methods:**

We could collect wound fluid samples from 4 fingertip injuries and 7 split skin donor sites at the 5th day during dressing change. Blood serum samples served as controls. The proliferation rate of MSCs and fibroblasts (HS27) was continuously measured through impedance analysis for 60 h and by Alamarblue analysis after 72 h. Cell migration was evaluated continuously for 15 h and confirmed by the in vitro wound-healing assay.

**Results:**

Migration of MSCs under the influence of both wound fluids was significantly faster than controls from 4 to 6 h after incubation and reversed after 9 h. MSC proliferation in wound fluid groups showed a significant increase at 5 and 10 h and was significantly decreased after 45 h. Fibroblasts in wound fluid groups showed overall a significant increase in migration and a significant decrease in proliferation compared to controls.

**Conclusion:**

OD-induced secretomes influence MSCs and fibroblasts and thereby possibly modulate wound healing and scar tissue formation.

## Background

Adult wound healing aims at rapidly closing a defect and reestablishing the skin barrier to prevent any further infection and fluid loss [1]. This process is commonly divided into at least three overlapping stages: inflammation, proliferation, and remodeling [2]. In inflammation, after initial hemostasis by the aggregation of platelets, the release of chemoattractants and growth factors recruits fibroblasts and leukocytes [2]. In proliferation, granulation tissue is predominantly formed by fibroblasts and macrophages, creating a new extracellular matrix, which allows epithelization, angiogenesis, and fibroplasia to take place [3]. Finally, in remodeling, collagen type III is replaced with collagen type I, resulting in a stable scar [3]. This whole process is reparative rather than regenerative, as the final scar tissue is a disturbance of the normal skin function, structure, and architecture with a reduced tensile strength by about 20–30% [4, 5].

In general, a scar is composed of excess extracellular matrix (ECM) compared to normal, uninjured dermal tissue and is devoid of epidermal appendages [5]. Purely regenerative healing of an inflicted wound in humans occurs in fetal wound healing up to 24 weeks of gestation [6]. It was demonstrated that fetal wounds differ from adult wounds in inflammatory response, gene expression, growth factor release as well as extracellular matrix production [7]. In scars, the extracellular matrix of the new dermis is abnormally deposited as small parallel bundles of collagen types I, III and fibronectin. In the non-injured skin, these bundles are large and appear in a basketweave orientation, suggesting a structural problem in the regenerative process [8]. Adult tissue loses the regenerative capacity and clinical problems owing to scars are manifold, including pathologies like keloids and hypertrophic scars, as well as symptoms like itching, pain or inhibition of movement due to contractions of joints [9].

However, also in adult wound healing, there are certain types of wounds which can heal nearly scar-free, resulting in a good aesthetic and functional outcome. Fingertip injuries treated with occlusive dressings (ODs) may regenerate up to 90% of the initial soft tissue loss, regenerate dermal ridges, and result in a good sensibility of the previously injured skin [10]. ODs create a moist wound environment and seem to play a key role for the favorable results seen in non-surgically treated fingertip amputations. As early as 1965, the effect of occlusive dressings was compared to air-exposed healing in human skin wounds by Hinman et al. [11]. In 1983, Soderberg et al. compared fingertip injuries treated with adhesive zinc tape dressings to surgical therapies. The authors found a better 2-point-discrimination sensitivity, as well as less scars and pain in patients treated with dressings alone [12]. Mennen and Wiese treated 200 patients with fingertip injuries with semi-occlusive dressings and reported excellent results, a near normal fingertip shape and a comparable sensitivity to the non-injured hand in between 20 and 30 days after the injury [13].

ODs allow the development of a moist wound environment, which immerses the cells and has been shown to increase collagen synthesis and to accelerate the rate of re-epithelialization [14, 15]. We hypothesized that the pivotal factor triggering such pro-regenerative stimuli underneath ODs is a direct effect of the wound fluid. This wound fluid is the sum of all biological substances, secreted from the sterile wound, and contains the secretome of the cells in the wound area. We have shown before that this secretome can change under wound conditions and can modulate wound-healing processes like angiogenesis [16–18].

In this study, we investigated the influence of such wound fluid from ODs on the behavior of mesenchymal stem cells (MSCs) and fibroblasts (FBs), important modulators of the wound-healing process.

## Methods

**Fig. 1:**
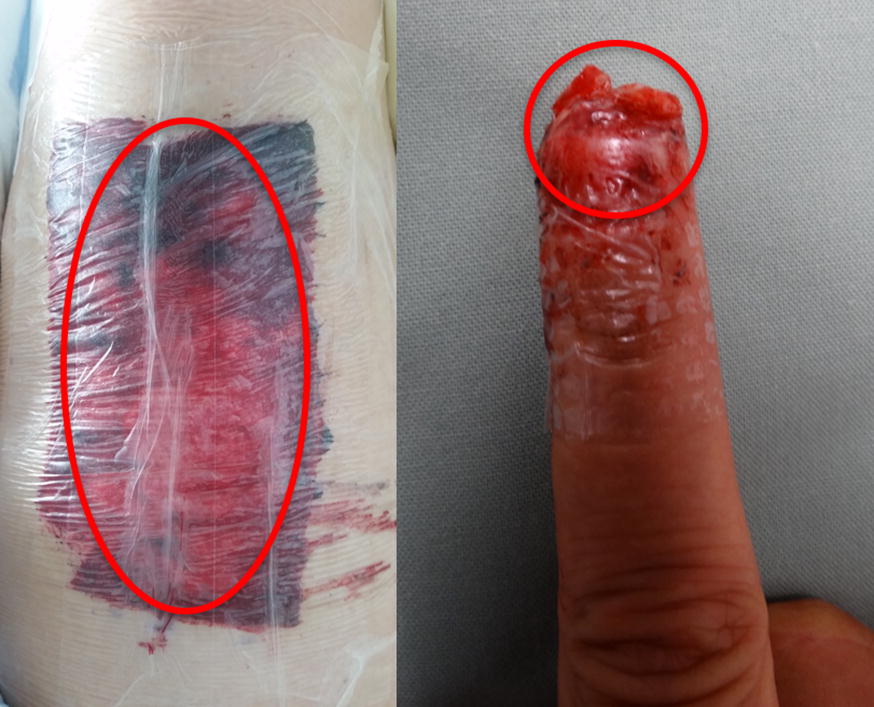
Wound fluid collection in a predesigned reservoir of an occlusive wound dressing of a patient with split skin donor site (left) and a fingertip amputation (right) (in the red circle)

The study protocol conformed to the guidelines of the 1975 Declaration of Helsinki as reflected in the approval of our local ethics committee (No. 527/15). 4 Fingertip injuries (Allen stages II–III) were treated with occlusive dressings (Smith & Nephew IV3000) after initial wound care. In addition, we treated 7 split skin donor site wounds (at the ventro-medial to ventro-lateral upper leg of the patients) with the same occlusive dressings postoperatively. At the 5th day, we tried to harvest fluid samples after obtaining informed consent from any patient while changing the dressings. This was done with a small syringe puncturing the dressing without injuring the patient’s skin. The fingertip injuries were dressed leaving a small reservoir at the top to allow a fluid collection, as is necessary for the functionality of the therapy (see Fig. 1) [19, 20]. We collected fluid from 4 fingertip injuries from 4 patients (minimum 60 µl, median 91 µl, and maximum 120 µl) and 7 split skin donor sites from 7 patients (minimum 3800 µl, median 2700 µl, and maximum 11,200 µl). Both patient groups had comparable demographics with an average age of 53 years in the fingertip group (range 52–55) and 55 years in the split skin group (range 39–82) without any comorbidities. The male-to-female ratio was 2:2 in the fingertip group and 2:5 in the split skin group.

Blood serum from each patient was drawn as controls (minimum 800 µl, median 2000 µl, and maximum 2500 µl). Due to limitations of fluid quantity per sample, we used 4 fingertip fluid samples and 4 of the 7 split skin fluid samples with respective blood serum controls in the Alamarblue Assay and xCELLigence analysis. The remaining 3 split skin donor site samples were analyzed in the in vitro wound-healing assay (Ibidi, Germany).

### Cell culture

HS-27, a commercially available human derived fibroblast cell line (ATCC—LGC Standards, Germany), was cultured in DMEM (Biochrom, Germany) supplemented with 10% FCS. HS-27 cells are human foreskin fibroblasts which are growing adherent to cell-culture plastic. We used adipose-derived MSCs also known as adipose-derived stem cells (ASCs or ADSCs) isolated from aesthetic liposuction, as previously described [21]. The cells were characterized in our lab by their expression of CD73, CD90, and CD105 as well as their potency to differentiate into fat cell, chondrocytes, and osteoblasts. For cultivation, MEM-alpha (Biochrom, Germany), containing 10% FCS was used.

### Cell metabolism assay (Alamarblue)

MSCs were seeded in a density of 8000 cells/well and HS-27 FBs in a density of 4000 cells/well in 96 well plates using standard cultivation media as described above. Cells were allowed to attach overnight, then the medium was replaced with sample (wound fluid or serum) in a concentration of 10%. Alamarblue assay (Sigma-Aldrich) was performed after 3 days of incubation at 37 °C in 5% CO_2_ atmosphere according to the instructions of the manufacturer. In short, Alamarblue reagent was added to the cells and incubated for 30 min, followed by fluorescence measurement at Ex 530 nm/Em 600 nm.

### Impedance proliferation assay (xCELLigence)

The xCELLigence device enables the continuous analysis of cell proliferation. The proliferation rates of MSCs and HS27 fibroblasts were determined with the xCELLigence-DP Analyser (OLS^®^ Omni Life Sciences, Germany). We seeded 4000 HS-27 FBs and 8000 MSCs in individual wells and incubated them with standard cultivation medium for 40 h. Wound fluid or serum was prediluted 1:10 in standard medium before it was added to the seeded wells in a ratio of 1:1 to achieve a final sample concentration of 5%. Continuous measurement of impedance (Cell index) started 30 min after seeding and was performed in intervals of 15 min for 100 h. Analysis was done for the first 60 h.

### Impedance migration assay (xCELLigence)

Migration analysis was performed in CIM plates in combination with xCELLigence-DP-Analyzer (OLS^®^ Omni Life Sciences). The CIM plate consists of an upper chamber with a porous membrane and a lower chamber. 40,000 HS-27 FBs or MSCs were seeded in standard medium in the wells of the upper chamber and left for 30 min to settle. As chemoattractant 5% wound exudate or 5% serum, diluted in standard medium, was filled in the wells of the lower chamber. Impedance measurement at the lower side of the porous membrane was carried out to visualize cell migration from the upper to the lower chambers. Impedance read out was expressed as cell index and executed every 15 min to a maximum of 25 h. Analysis was done for the first 15 h.

### In vitro wound-healing assay (Ibidi)

We first pipeted 70 μl cell suspension (1.2 × 10^5^ cells/ml) in each of the 24 wells of Ibidi culture inserts (Ibidi, Germany). Next, cells were incubated at 37 °C and 5% CO_2_ for 24 h to obtain a confluent cell layer. Culture inserts were removed afterwards and cell layers washed with PBS. Filled plate wells were prepared with culture medium (Dulbecco’s MEM by Merck Millipore, 2% wound fluid and 1% antibiotic/antimycotic ingredient by Capricorn, AAS-B) up to 500 μl. Wells filled with medium without wound fluid were used as negative controls. Microscopy pictures were taken at 0 h, 6 h, 12 h, and 24 h and images were analyzed using the WimScratch software.

### The statistical analysis

Data are shown as mean ± SEM. Statistical analysis between the groups was performed using unpaired *t* tests. *p* values of less than 0.05 were considered statistically significant.

## Results

### Migration assay (xCELLigence impedance)

**Fig. 2:**
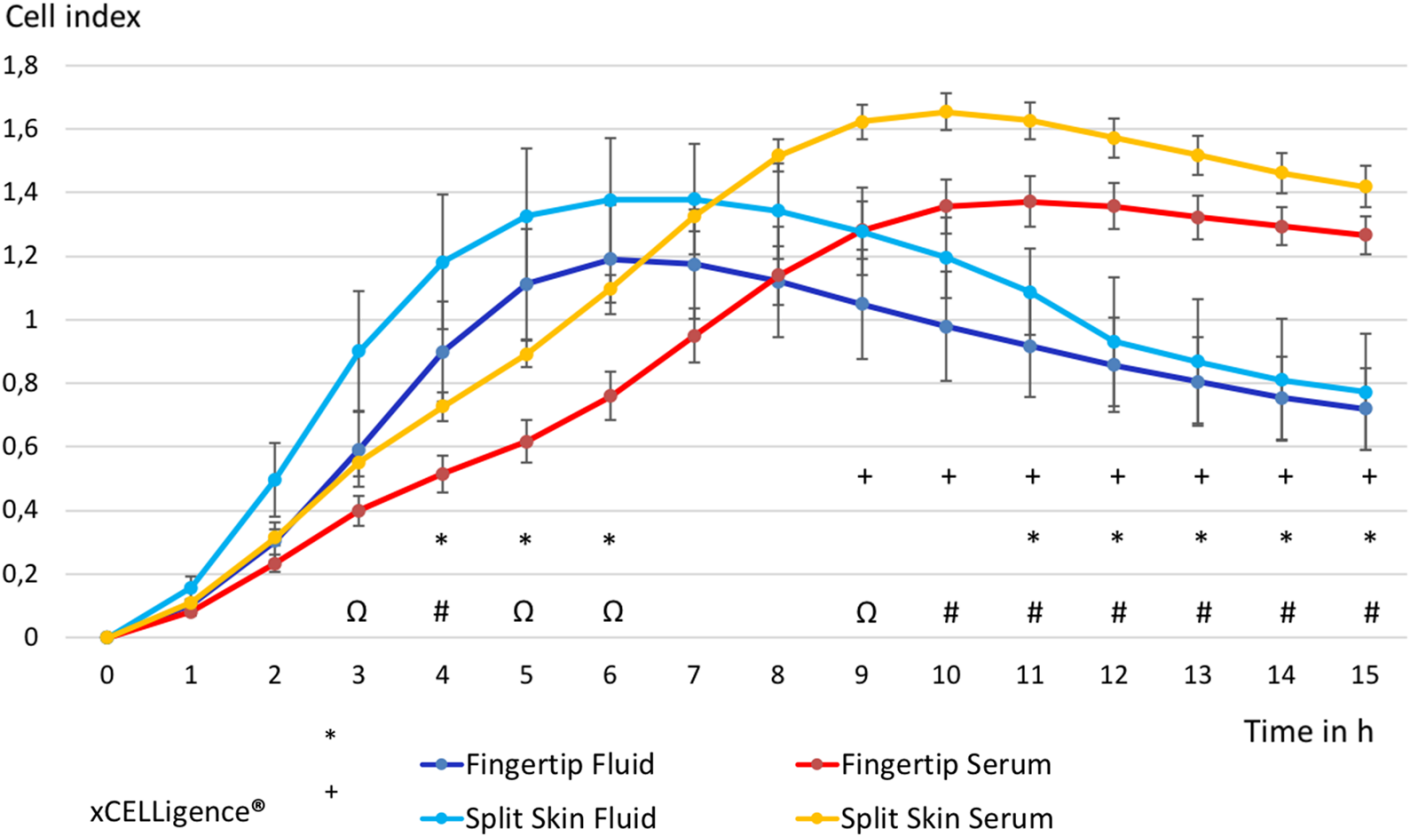
Time-dependent MSC migration in wound fluid groups vs. control groups. All groups in mean ± SEM. *p < 0.05 mean fingertip fluids vs. mean fingertip serums. ^+^*p* < 0.05 mean split skin fluids vs. mean split skin serums. ^#^*p* < 0.01, ^Ω^*p* < 0.05 mean all fluids vs. mean all serums

The migration of MSCs under the influence of fingertip fluid measured with xCELLigence was significantly increased at 4 h (*p* = 0.046), 5 h (*p* = 0.032) and 6 h (*p* = 0.048) compared to blood serum controls of the same patients. This effect reversed significantly in the MSC population of occlusive treated fingertip injuries after 11 h (*p* = 0.047) and continued afterwards (see Fig. 2). MSCs under the influence of split skin fluid showed initially a trend towards an increased migration compared to respective serum controls, which was not significant. A significant decrease of MSC migration, influenced by split skin donor site fluid, was found starting at 9 h after incubation (*p* = 0.049) and continued to do so (see Fig. 2). Combined analysis of MSC migration of both wound fluids compared to both serum controls showed a significant increase from 3 h (*p* = 0.023) to 6 h (0.017) with the strongest increase at 4 h (*p* = 0.001). A significant decrease in MSC migration of all fluids compared to all serum values started at 9 h (*p* = 0.034) onwards (see Fig. 2).

**Fig. 3:**
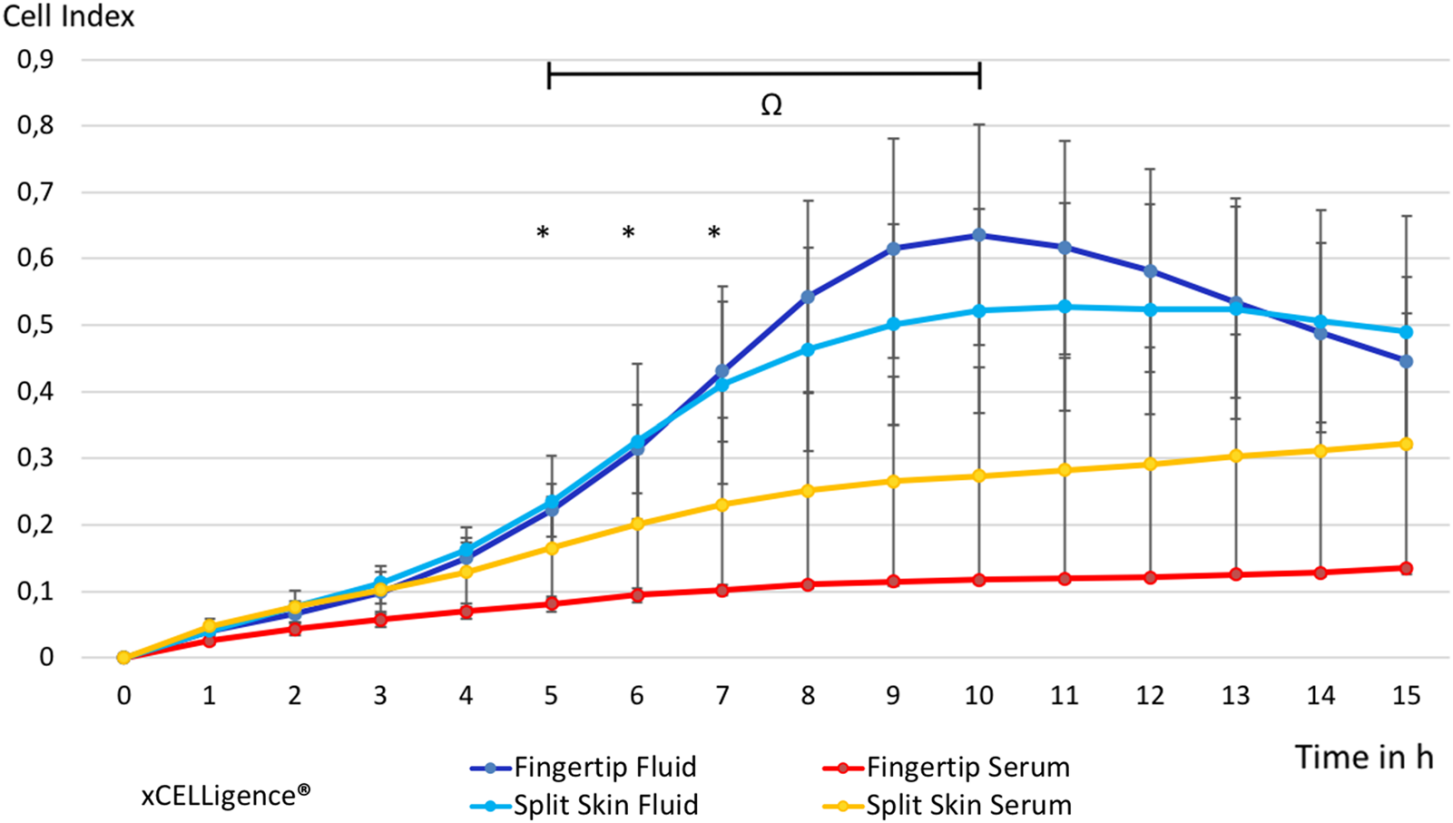
Time-dependent HS27 FB migration in wound fluid groups vs. control groups. All groups in mean ± SEM. *p < 0.05 mean fingertip fluids vs. mean fingertip serums. ^#^*p* < 0.01 mean of all fluids vs. mean all serums

The migration of HS27-FBs of fingertip fluids in xCELLigence was significantly increased at 5 h (*p* = 0.041), 6 h (*p* = 0.043) and 7 h (*p* = 0.049) compared to respective serum controls (see Fig. 3). The migration of HS27 FBs of split skin fluid alone did not show any significant changes, but mimicked the curve of the fingertip fluid values with a less steep increase and decline after a few hours. Analyzing the migration HS27 FBs of all wound fluid samples compared to all serum samples showed a significant increase between 5 and 10 h after sample incubation (*p* = 0.004) (see Fig. 3).

### In vitro wound-healing assay

**Fig. 4:**
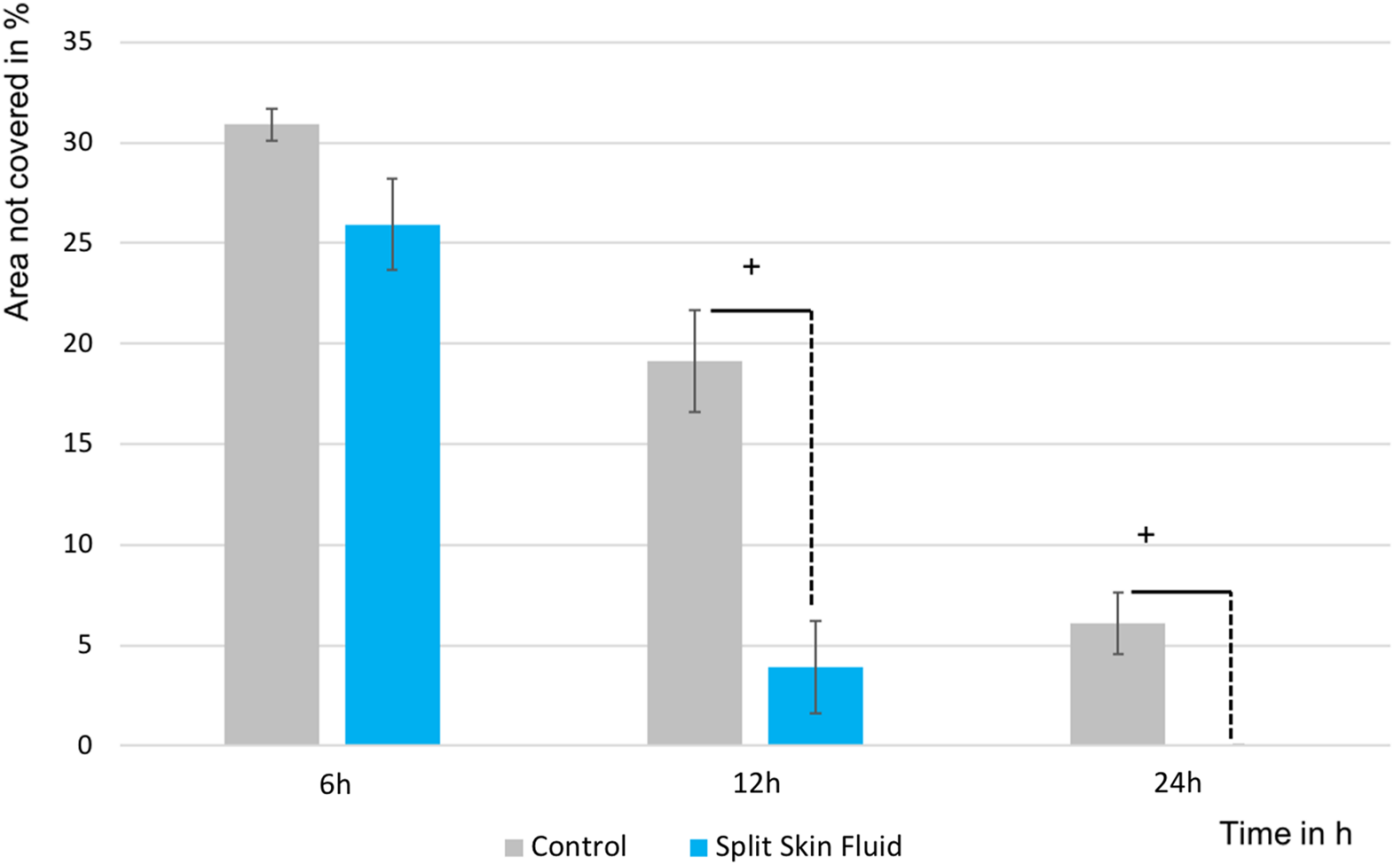
HS27 FB migration in the in vitro wound-healing assay. All groups in mean ± SEM. ^+^*p* < 0.05 mean split skin fluids vs. mean controls

**Fig. 5:**
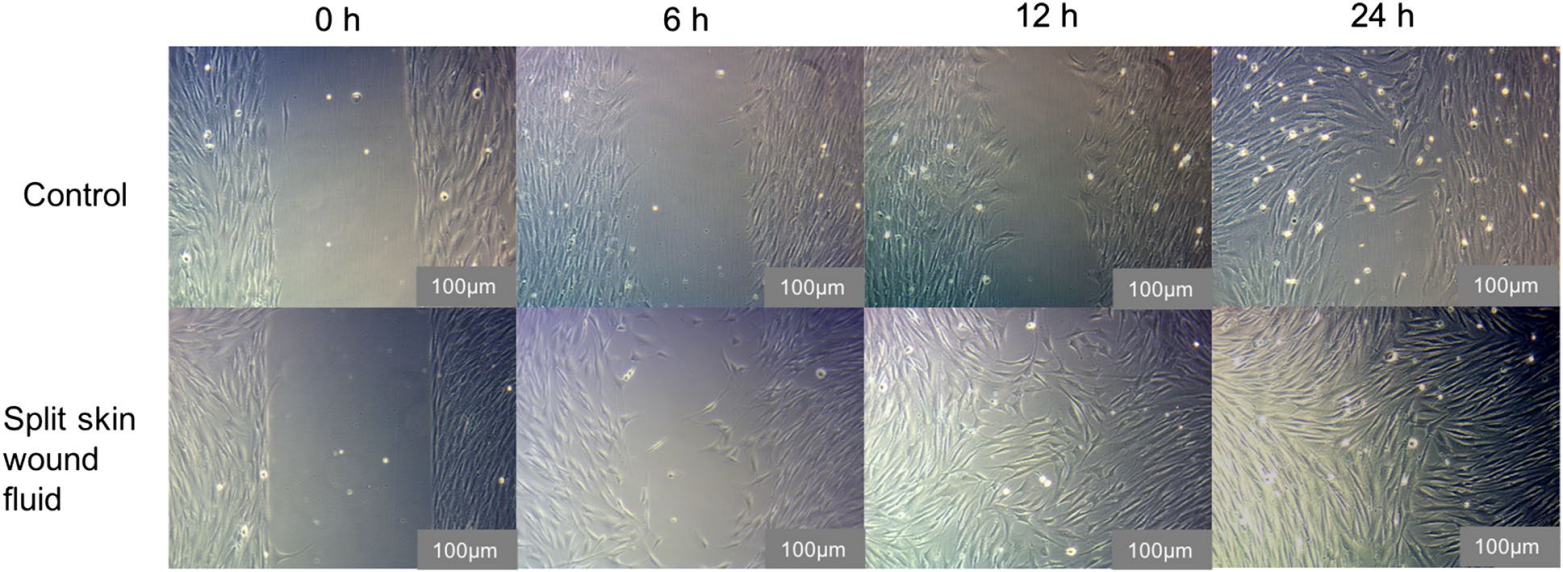
Representative microscopic images of cell migration at different time points in the in vitro wound-healing assay using 100-times magnification (scale bar size: 100 μm)

The in vitro wound-healing assay showed a significantly increased HS27 FB migration at 12 h (*p* = 0.012) and at 24 h (*p* = 0.017) compared to the control, supporting our data from the impedance analysis (see Figs. 4, 5).

### Proliferation I (xCELLigence impedance)

**Fig. 6:**
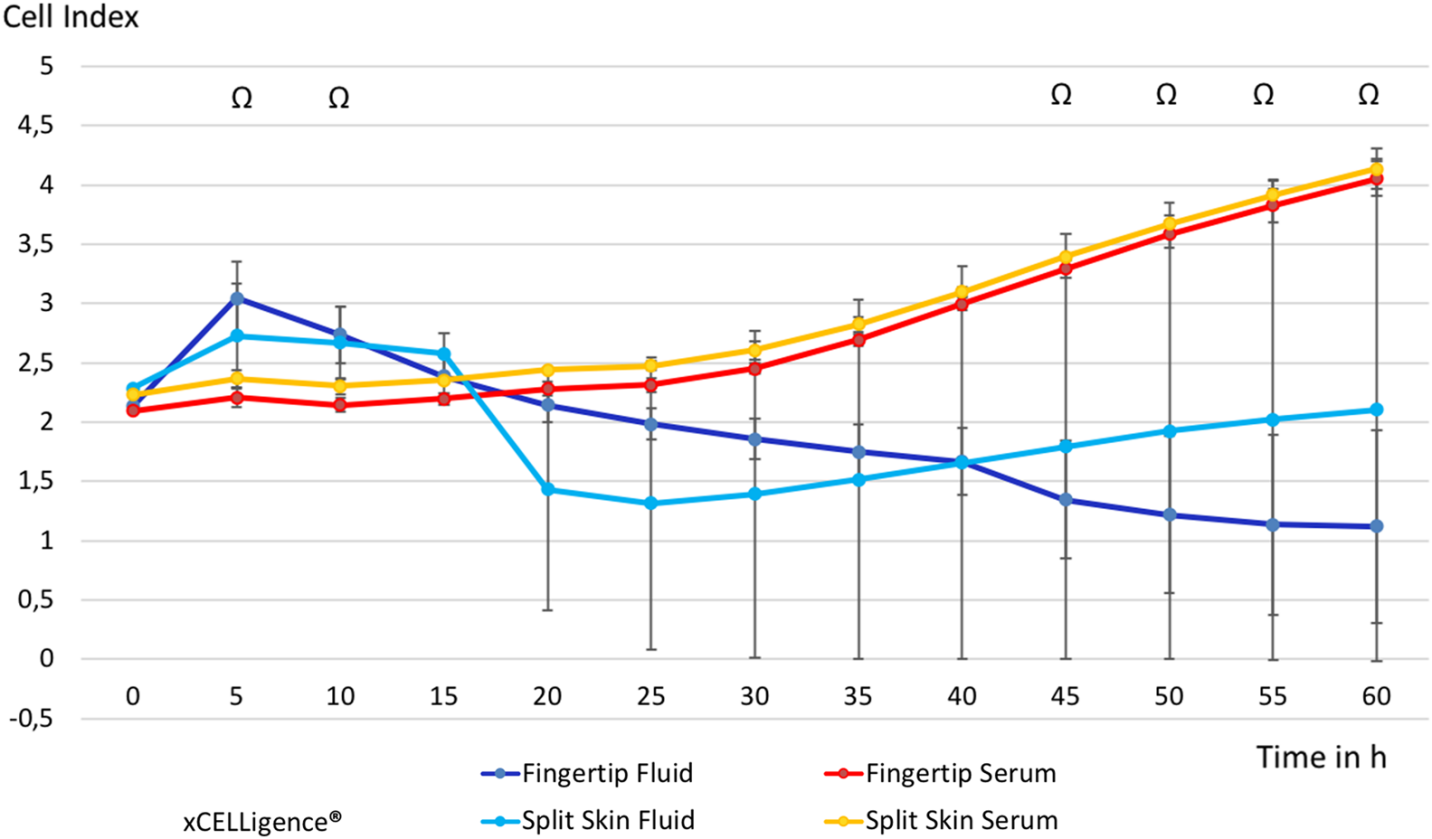
Time-dependent MSC proliferation in wound fluid groups vs. control groups. All groups in mean ± SEM. ^Ω^p < 0.05 mean all fluids vs. mean all serums

**Fig. 7:**
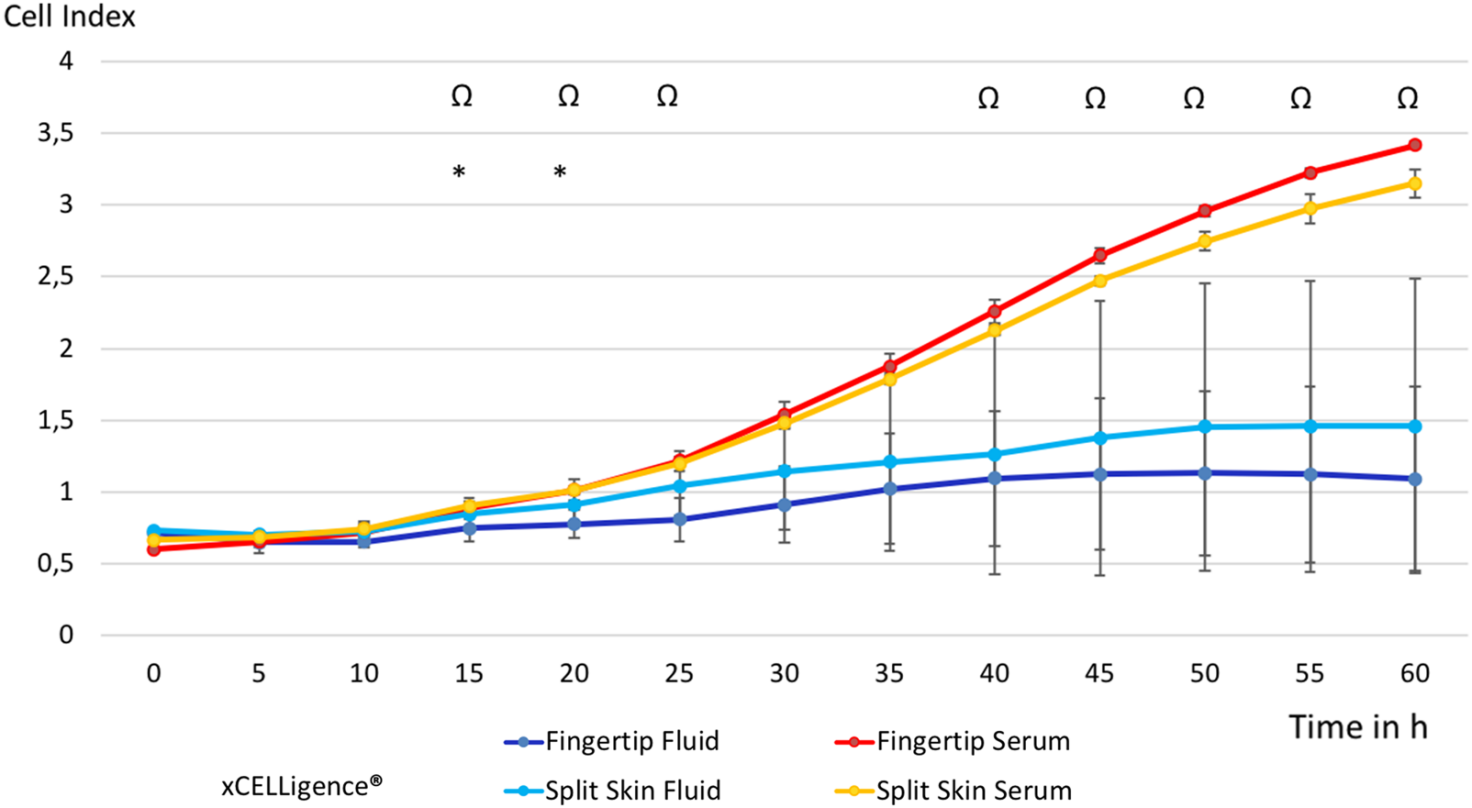
Time-dependent HS27 FBs proliferation in wound fluid groups vs. control groups. All groups in mean ± SEM. *p < 0.05 mean fingertip fluids vs. mean fingertip serums. ^Ω^*p* < 0.05 mean all fluids vs. mean all serums

We observed a non-significant increase of cell proliferation of MSCs exposed to each fluid alone at 5 h (fingertips *p* = 0.061, split skin donor sites *p* = 0.511) and 10 h (fingertips *p* = 0.091, split skin donor sites *p* = 0.511). By comparing of all fluid samples with the respective serum samples, we observed a significant increase at 5 h (*p* = 0.034) and 10 h (*p* = 0,041). However, this effect was reversed at 15 h. A significant decrease in MSC proliferation of both wound fluids combined compared to the controls was seen starting at 45 h of incubation (*p* = 0.046) and later (see Fig. 6). The proliferation of HS27 FBs showed initially a slow decrease in both fluids, which was significant in the fingertip fluid group compared to the control group at 15 h (*p* = 0.012) and 20 h (*p* = 0.030). The pooled analysis of HS27 FB proliferation of all fluids compared to all serum controls was significantly decreased from 15 h (*p* = 0.022) to 25 h (0.039), continuing to be so at 40 h (*p* = 0.044) onward (see Fig. 7).

### Cell metabolism/proliferation II (Alamarblue)

**Fig. 8:**
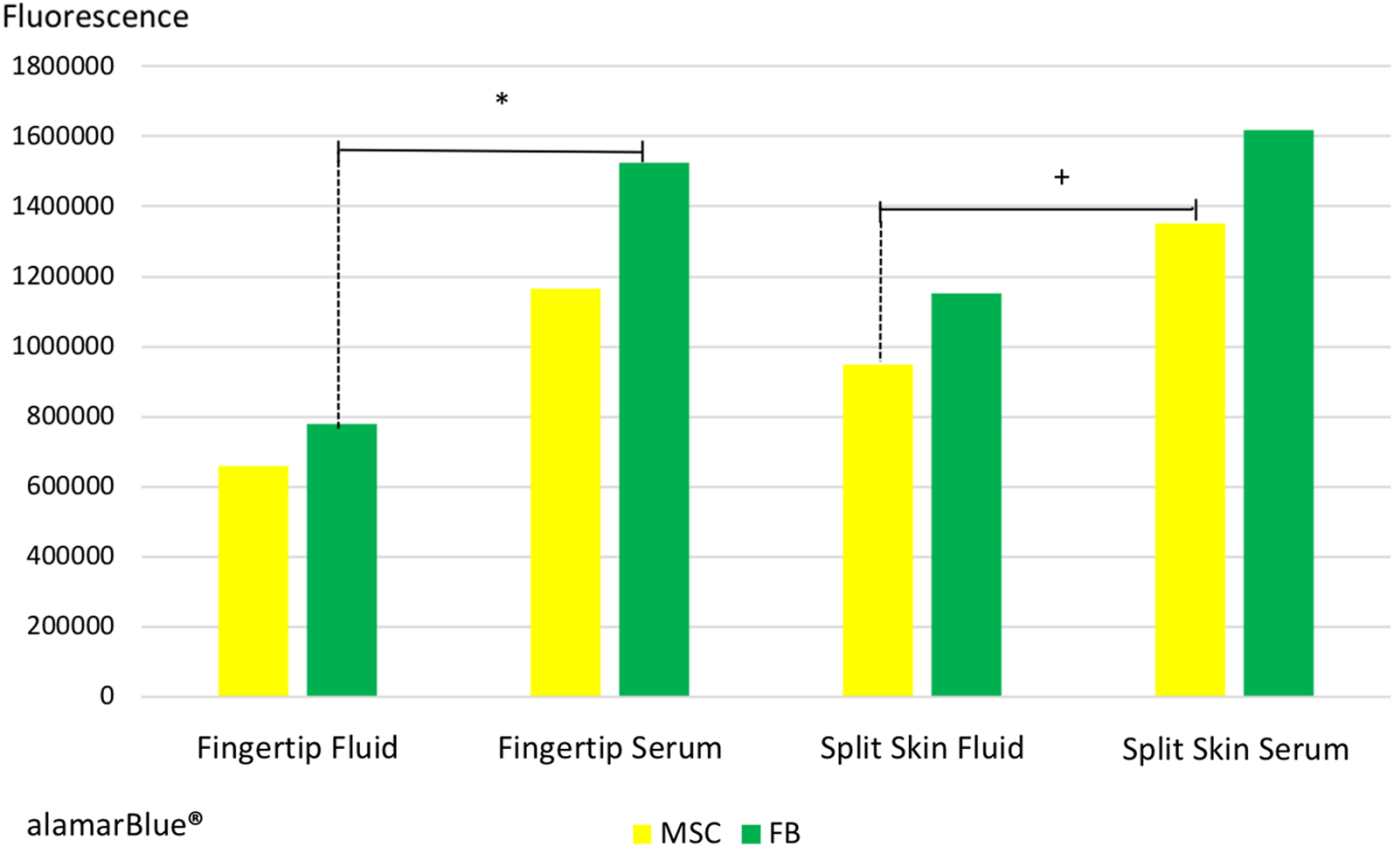
Proliferation of MSCs and HS27 FBs after 3 days of sample incubation. All groups in mean ± SEM. *p < 0.01 mean fingertip fluids vs. mean fingertip serums. ^+^*p* < 0.05 mean split skin fluids vs. mean split skin serums

HS27 FBs were significantly reduced in the fingertip fluid group compared to respective controls (*p* = 0.006), whereas MSCs were significantly decreased only in the split skin fluid group compared to controls (*p* = 0.0448). We saw a significant decrease in MSC proliferation exposed to both combined wound fluids compared to blood serum controls (*p* = 0.008). HS27 FB proliferation also decreased in both pooled fluid groups significantly compared to the controls (*p* = 0.003) after 3 days of sample incubation (see Fig. 8).

We did not observe any statistically significant difference in the behavior of mesenchymal stem cells and fibroblasts between samples from fingertip or split skin donor site wound fluids.

## Discussion

A moist wound environment stimulates fibroblast proliferation, enhances the expression of growth factors, and induces angiogenesis, which finally leads to a faster regeneration [22, 23]. In addition, the formation of scars, as well as scar quality in general is improved, achieving a better functional and aesthetic outcome [24]. These effects on scars might be due to a shorter inflammation and proliferation phase in moist dermal wound healing compared to dry dressing changes [25]. In line with this hypothesis, Ksander et al. demonstrated that moisture vapor-permeable dressings inhibited the deposition of granulation tissue and collagen compared to air-exposed wounds in animal models. Histologic results of these wounds showed large amounts of fibrous connective tissue with mononuclear and polymorphonuclear cells in air-exposed wounds, whereas wounds treated with moist dressings showed a lack of these tissues and cells as well as decreased inflammation [26]. O’Shaughnessy et al. demonstrated that occlusion reduces epidermal water loss and may restore the homeostasis of the epidermal barrier, which decreases hypertrophic scarring and inflammation-specific cells like macrophages [27]. However, the interplay of FBs and MSCs in moist wound healing and the influence of wound exudate on local cells in the wound bed is incompletely understood.

The formation of granulation tissue and cellular interactions in the inflammatory and proliferation phases seems to differ significantly in moist wound healing compared to air-exposed wounds [25, 26, 28]. Fibroplasia and the creation of granulation tissue are mainly dependent on fibroblasts, which are the dominant cell type by day 4 of wound healing and peak at 7–14 days after injury [29, 30]. Platelet-derived growth factor (PDGF), basic fibroblast growth factor (bFGF), and transforming growth factor-β (TGF-β) modulate the initial migration and proliferation of fibroblasts, which then reorganize and replace the provisional matrix through synthesis and the release of collagen [31, 32]. Finally, in remodeling which takes up to 2 years, the provisional matrix is restructured by matrix metalloproteinases to increase tensile strength, resulting in a scar [3].

As important as fibroblasts are for the formation of the proliferative tissue, the production of collagen and finally its differentiation into myofibroblasts to allow wound contraction is mainly induced by MSCs, which are the important modulators every step of the way [33]. In the inflammatory phase, MSCs act as anti-inflammatory agents by increasing anti-inflammatory cytokines like interleukin-10 (IL-10) and IL-4, as well as prohibiting the secretion of pro-inflammatory cytokines like TNF-α and Interferon-γ [34]. In chronic wounds, all wound-healing phases are prolonged, with immense infiltration by neutrophil granulocytes [35]. MSCs can interrupt this cycle and allow wound healing by decreasing the inflammatory state, by promoting cell migration and proliferation as well as by induction of angiogenesis [36]. Our results demonstrate a trend towards an increase in wound-healing quality, where a strong migration of MSCs and fibroblasts possibly allow the multiple tasks of these cells to take place, but limiting an overabundance of fibroblasts, which is known to be found in hypertrophic scars [4].

MSCs act predominantly by paracrine signaling and can secrete cytokines and growth factors like PDGF, vascular endothelial growth factor (VEGF), epidermal growth factor (EGF), bFGF, keratinocyte growth factor (KGF), and TGF-β, thus modulating the proliferative phase of wound healing [36]. Studies showed that medium conditioned with MSCs or MSCs alone induces dermal fibroblasts to increase the rate of wound closure and that MSCs stimulate the proliferation of fibroblasts, endothelial cells, and keratinocytes in vitro [37]. In response to MSC stimulation, collagen type I is increased by dermal fibroblasts, thus accelerating wound closure [37]. Taken these findings together, MSCs may directly influence a decrease in scar formation by inducing organized extracellular matrix (ECM) deposition [33].

### Scar and wound modulation by occlusive dressings

Recent studies showed that hypoxic stimuli seem to mobilize MSCs from the wound bed and that continuous hypoxia increases MSC mobilization by supposedly upregulation of hypoxia-induced factor-1α (HIF-1α) [38]. In our study, we demonstrated a significant increase of MSC mobilization when exposed to fluid from OD wounds, possibly showing one more factor involved in modulating wound repair. Here, we observed a stronger increase of MSC migration in the fingertip fluid group as in the occlusive-dressed split skin fluid group. As MSC motility decreases after reaching a plateau in our experiments, there seem to be further mechanisms in the regulation of MSC activity involved. Occlusive dressings, which maintain a hypoxic wound environment, could be responsible for the observed positive effects on wound healing [14, 15].

It would have been very interesting to study the contents of the secretome in our wound fluids in more detail, but unfortunately, we were restricted by the amount of fluid that could be obtained from our patients. Per patient, we only could obtain around 90 µl of fluid, so we had to decide to either study the proteomic content or do cell-culture analysis. Here, we decided to focus on the cell behavior modulation of fibroblasts and MSCs. Fortunately, there are already data available about the contents of such wound fluids: Vogt et al. analyzed wound fluid from occlusive-dressed fingertip injuries on growth factors and found upregulated bFGF, epidermal growth factor (EGF), interleukin-1α (IL-1α), and TGF-β2 compared to blood serum values, which could be a reason why ODs on fingertip injuries show a strong regenerative potential [39]. It was hypothesized that wounds heal faster due to fibroblast and keratinocyte proliferation, increased angiogenesis, and growth factor expression [22, 23]. As fibroplasia and the formation of granulation tissue mainly bases on an initial burst of fibroblast activity, scar quality should not be increased due to an overabundance of proliferating fibroblasts, which are often seen in hypertrophic scars [35]. Our results show that fibroblast proliferation seems to be inhibited by occlusive-dressed wound fluids, which could be another major factor to initiate a favorable scar modulation process. These data are corroborated by a recent study, showing that inhibiting a known-scar-forming fibroblast lineage during wound healing did not lead to a change of healing rates during the initial days of wound healing. However, a significant difference in wound size developed by day 9 after wounding, with larger and less healed wounds in the treated cohorts. Most importantly, treated wounds showed significantly reduced final scar size after completion of wound healing [40]. Similarly, we could observe a spike of fibroblast mobility in the first 10 h after sample incubation, which afterwards decreased significantly. As fibroblasts are important players in the wound-healing process, their active role in the inflammatory and proliferative phase of wound healing is essential [35]. In dry wound healing, a fast wound closure is important to minimize fluid loss and reduce the risk of infection, thus an increased fibroblast proliferation and activity is necessary to minimize these risks, but leading to a scar [1]. A superficial occlusive barrier, allowing wound fluid to cover the wound, seems to inhibit excessive fibroblast proliferation, as we could show in our study. Interestingly, this effect was more pronounced in the fingertip fluid samples as in the split skin fluid samples, which could be responsible for the good clinical outcomes observed in occlusively dressed fingertip injuries. In addition, split skin fluids showed a significantly decreased MSC proliferation after 3 days, which was not that prominent in the fingertip fluid group, suggesting that also other factors contribute to the positive effects of ODs in wound healing [25].

### Limitations

Our results were generated in a simplified in vitro model of cell migration and proliferation. This is limited by several factors: the wound fluid is only administered once in the model, while it is presumably constantly produced in the in vivo wound situation. Thus, the results in the early hours of the experiment may reflect the wound environment better than the later data. In vivo, the wound is an injured 3D-tissue consisting of extracellular matrix and a multitude of cells that constantly interact with each other through autocrine, paracrine, electrical, and mechanical cues that are not reproduced in the model. On the other hand, this approach allowed us to isolate effects of the wound fluid on two specific cell types that are thought to be important in wound healing, which is not as easily possible in a more complex model.

## Conclusion

Using advanced cell surveillance technology, we could demonstrate a time-dependent and cell-type-related effect of wound fluid on migration and proliferation of mesenchymal stem cells and fibroblasts. This time-resolved information revealed cell behavior that is nearly impossible to study in endpoint measurements. MSCs are important modulators of the proliferation stage in wound healing and play an important role in the formation of scar tissue. The early activation of MSC migration could modulate initial tissue composition and thereby scar tissue formation. Fibroblast proliferation seems to be inhibited in occlusively dressed wounds and may improve the organization of collagen structure on the wound. This well-orchestrated interplay between stromal cells may be partially responsible for the improved wound-healing process and scar quality, which are seen in fingertip injuries and split skin donor sites treated under occlusive conditions.

## Abbreviations

ECM: extracellular matrix
ODs: occlusive dressings
MSCs: mesenchymal stem cells
ASCs or ADSCs: adipose-derived stem cells
